# Comparative Analysis of Streptococcus mutans and Lactobacillus spp. Colonization on Stainless Steel versus Zirconia Crowns in Root Canal-Treated Teeth

**DOI:** 10.7759/cureus.91450

**Published:** 2025-09-02

**Authors:** Bahni S Pathak, Alpana Talukdar, Indrani Barman, Shivansh Aggrohiya, Upasana Barman, Samar Khan, Seema Gupta

**Affiliations:** 1 Department of Dentistry, Dhubri Medical College and Hospital, Dhubri, IND; 2 Department of Dentistry, Nalbari Medical College and Hospital, Nalbari, IND; 3 Department of Dentistry, Pragjyotishpur Medical College and Hospital, Guwahati, IND; 4 Department of Conservative Dentistry and Endodontics, Faculty of Dental Sciences, Shree Guru Gobind Singh Tricentenary University, Gurugram, IND; 5 Department of Pedodontics and Preventive Dentistry, Dent-O-Shine Dental Clinic, Guwahati, IND; 6 Department of Conservative Dentistry and Endodontics, People's College of Dental Sciences and Research Centre, Bhopal, IND; 7 Department of Orthodontics, Kothiwal Dental College and Research Centre, Moradabad, IND

**Keywords:** dental crowns, lactobacillus, root canal therapy, streptococcus mutans, zirconia

## Abstract

Introduction: Root canal-treated teeth are often restored using crowns to maintain function and protect against damage. The choice of crown material, such as stainless steel or zirconia, may influence microbial colonization and potentially affect restoration longevity. Bacteria commonly found in the oral environment, such as *Streptococcus mutans* and *Lactobacillus* spp., may adhere to crown surfaces and form biofilms. Differences in surface properties such as roughness and hydrophilicity may affect bacterial adhesion and biofilm formation. This study aimed to quantify and compare the microbial loads of *S. mutans* and *Lactobacillus* spp. on stainless steel and zirconia crowns in RCT-treated teeth and to characterize the species diversity within biofilms formed on these surfaces.

Materials and methods: This prospective, non-randomized, clinical study enrolled 20 patients requiring crowns on root canal-treated lower first molars using a split-mouth design. The patients received stainless steel (3M ESPE AG, Seefeld, Germany) and zirconia (VITA Zahnfabrik, Bad Säckingen, Germany) crowns based on clinical indications and preferences. Oral prophylaxis was performed using ultrasonic scalers (E.M.S. Electro Medical Systems S.A., Nyon, Switzerland), and crowns were placed within 14 days of RCT by using an endodontic system (Dentsply Sirona Inc., Charlotte, North Carolina, United States). Impressions were made with light body polyvinyl siloxane putty (Elite HD+; Zhermack SpA, Badia Polesine, Italy), and marginal fit was verified clinically. Microbial samples were collected at baseline, one month, and six months using sterile paper points, and the gingival and plaque indices were recorded. Quantitative polymerase chain reaction (qPCR) using a LightCycler 480 System (Roche Diagnostics Corporation, Indianapolis, Indiana, United States) was used to quantify *S. mutans* and *Lactobacillus* spp. Data were statistically analyzed.

Results: Both crown types showed significant increases in plaque index, probing depth, *S. mutans*, and *Lactobacillus* spp. over time (p = 0.001). Stainless steel crowns exhibited significantly greater increases in all parameters than zirconia crowns (p < 0.05), with the largest difference in *Lactobacillus* spp. colonization.

Conclusion: Zirconia crowns demonstrated lower microbial colonization than stainless steel crowns, suggesting greater biocompatibility for root canal-treated teeth. These findings support zirconia as the preferred material for minimizing microbial-related complications in restorative dentistry.

## Introduction

Root canal treatment (RCT) is a widely performed endodontic procedure aimed at eliminating infection from endodontic periapical lesions and preserving the functionality of teeth with compromised pulp [1]. It is widely acknowledged that teeth subjected to endodontic therapy exhibit a heightened susceptibility to fracture compared to untreated teeth; however, the primary determinant influencing their structural integrity seems to be the quantity of residual natural tooth material [2]. Furthermore, endodontic procedures typically necessitate the excision of certain portions of the tooth structure, which may compromise the strength of the remaining dental tissue [3]. Therefore, crowns are often placed following RCT to restore structural integrity and protect the treated tooth from further damage [4].

The choice of crown material, metal or ceramic, has been a subject of extensive debate, primarily because of differences in aesthetics, mechanical properties, and biocompatibility [5]. However, an underexplored yet critical aspect is the susceptibility of these materials to microbial colonization, which can influence the long-term success of root canal-treated teeth. Microbial colonization of dental restorations is a significant concern, as it can lead to secondary infections, periapical lesions, or failure of endodontic treatment [6]. Dental crowns are in constant contact with the oral environment and are exposed to diverse microbial flora, including *Streptococcus mutans*, *Lactobacillus *spp., and *Porphyromonas gingivalis*, which are implicated in dental caries and periodontal diseases [7].

The surface properties of restorative materials, such as roughness, hydrophobicity, and chemical composition, play pivotal roles in microbial adhesion and subsequent biofilm development [8]. Metal crowns, typically made from alloys such as cobalt-chromium or nickel-chromium, are known for their durability but may exhibit different surface characteristics compared to ceramic crowns, which are composed of materials such as zirconia or lithium disilicate and are favored for their esthetic appeal [6]. These differences may influence the extent and nature of microbial colonization, potentially affecting the prognosis of the root canal-treated tooth.

Previous research suggests that esthetic crowns, such as zirconia, owing to their smoother surfaces and lower surface free energy, may resist bacterial adhesion more effectively than metal crowns [6,7]. Bin AlShaibah et al. reported significantly increased bacterial adhesion of *S. mutans* on preveneered crowns compared to that on stainless steel crowns [9]. Furthermore, the oral environment of root canal-treated teeth, often characterized by altered salivary flow or compromised immune responses, may exacerbate microbial colonization, making the choice of crown material critical.

This comparative study evaluated the microbial load, species diversity, and biofilm characteristics of *S. mutans* and *Lactobacillus *spp. on stainless steel and zirconia crowns in root canal-treated teeth. This study aimed to quantify and compare the extent of microbial colonization and biofilm formation on crown materials using advanced microbiological techniques such as polymerase chain reaction (PCR). The objectives of this study were to measure the microbial load of *S. mutans* and *Lactobacillus *spp. on stainless steel and zirconia crowns and to characterize the species diversity within biofilms formed on these surfaces. These findings provide insights into the biocompatibility of stainless steel and zirconia crowns in endodontic treatment, guiding clinicians in selecting materials that minimize microbial-related complications and contribute to improved clinical outcomes in restorative dentistry.

## Materials and methods

This was a comparative clinical study, designed as a prospective non-randomized study, conducted at the Department of Conservative Dentistry and Endodontics, People's College of Dental Sciences and Research Center, Bhopal, Madhya Pradesh, India. The study spanned from October 2021 to March 2023, and encompassed patient recruitment, crown placement, follow-up, and microbial analysis. Ethical approval was obtained from the Institutional Ethics Committee (IEC) of People's College of Dental Sciences and Research Center (IEC project code: EC202116), and all procedures complied with the Declaration of Helsinki (2013 revision). Written informed consent was obtained from all patients after providing detailed information about the study’s purpose, procedures, risks, and benefits, with assurance of their right to withdraw at any time without affecting their dental care.

Sample size calculation

The sample size was calculated using G*Power software (version 3.1.9.2, Heinrich-Heine-Universität Düsseldorf, Düsseldorf, Germany) with 80% power and 5% alpha error. Based on an effect size of 0.57 (derived from prior research comparing *S. mutans* levels between metal and ceramic crowns), a minimum of 20 patients per group was required [7]. The split-mouth design enabled this to be achieved in 20 patients. 

Eligibility criteria

Eligible patients were aged >18 years, required crowns on root canal-treated lower first molars on both sides, and had no systemic disease, active periodontal disease, or allergies to dental materials. The exclusion criteria were recent antibiotic use, smoking, and pregnancy. A total of 20 patients who received stainless steel crowns (3M ESPE AG, Seefeld, Germany) and zirconia crowns (VITA Zahnfabrik, Bad Säckingen, Germany) were included in the study. Only patients with RCT completed within three months prior to crown placement were included to minimize variability in the oral environment due to coronal leakage or secondary infections. This was verified using the patient records and clinical examinations.

**Figure 1:**
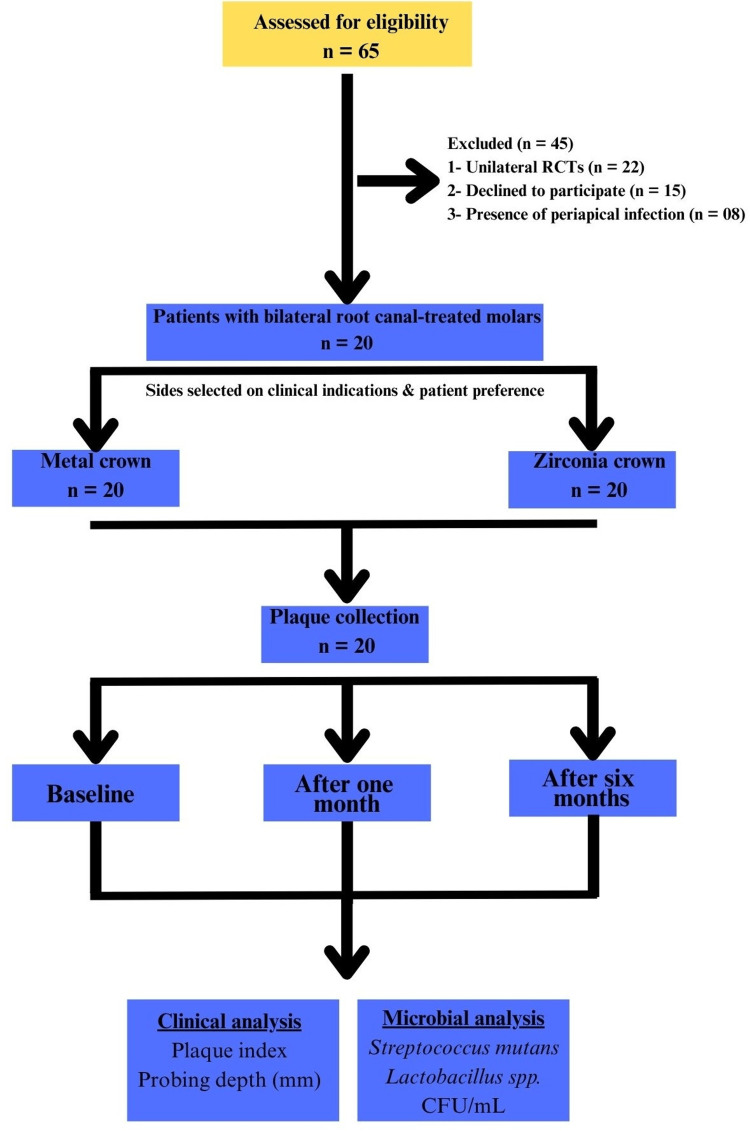
Study flowchart. RCT: root canal treatment, CFU/mL: colony-forming units per milliliter.

Patients requiring crowns on bilateral root canal-treated teeth were assigned to receive stainless steel or zirconia crowns on either side based on clinical indications and patient preference (Figure 1).

Crown placement

Prior to crown placement, all patients underwent oral prophylaxis using ultrasonic scalers (E.M.S. Electro Medical Systems S.A., Nyon, Switzerland) to remove plaque and calculus, ensuring a standardized baseline oral hygiene. After RCT completion using an endodontic system (Dentsply Sirona Inc., Charlotte, North Carolina, United States), temporary restorations (Cavit; 3M ESPE AG) were placed, and crowns were fitted within 14 days to minimize coronal leakage. Proper impressions of the crowns were obtained using light-body polyvinyl siloxane putty (Elite HD+; Zhermack, Badia Polesine, Italy) to ensure an accurate fit. Crown preparations for RCT-treated lower first molars were standardized, involving 1-1.5 mm axial and 1.5-2 mm occlusal reduction with a 2 mm ferrule height, using high-speed diamond burs (Komet Dental, Lemgo, Germany) under water cooling. Stainless steel crowns were fabricated via lost-wax casting, while zirconia crowns were milled using computer-aided design/computer-aided manufacturing (CAD/CAM) (CEREC®, Dentsply Sirona Inc.). Finishing was performed with fine diamond burs, followed by polishing with rubber cups and pastes (Shofu Super-Snap; Shofu Inc., Kyoto, Japan) to achieve a smooth surface, minimizing microbial adhesion.

The fit of the stainless steel and zirconia crowns was clinically verified by calibrated dentists. The crown margins were examined under high-intensity dental operatory light using dental loupes (Orascoptic, Madison, Wisconsin, United States) with 3.5 × magnification to assess the crown-tooth interface for gaps, overhangs, or irregularities. Dental floss (Oral-B Glide; The Procter & Gamble Company, Cincinnati, Ohio, United States) was passed through the interproximal areas to check the marginal fit. The floss was gently inserted to evaluate resistance, frailty, or catching at the crown margin, with a smooth passage indicating good adaptation.

Patients were provided with standardized oral hygiene instructions, including twice-daily brushing with fluoride toothpaste (Colgate-Palmolive Company, New York, United States) and daily flossing, to minimize the differences in hygiene practices. Compliance was monitored through patient self-reports during follow-up visits. All patients were advised to follow a standardized low-sugar diet throughout the study period.

Sample collection

Microbial samples were collected from the crown surfaces at three time points: immediately after crown placement (T0), at one month (T1), and at six months post-placement (T2), using sterile paper points (Dentsply Sirona Inc.) under aseptic conditions. Additionally, gingival and plaque indices were recorded at these time points to assess periodontal health and oral hygiene [10].

The samples were transported to the laboratory in sterile saline within two hours. DNA extraction was performed using the QIAamp DNA Mini Kit (QIAGEN, Hilden, Germany), followed by quantitative polymerase chain reaction (qPCR) to quantify *S. mutans* and *Lactobacillus* spp. using specific primers and a LightCycler 480 system (Roche Diagnostics Corporation, Indianapolis, Indiana, United States). Microbial load was quantified as colony-forming units per milliliter (CFU/mL) based on standard curves generated from known bacterial concentrations. This method provides high sensitivity and specificity for detecting and quantifying target bacteria, allowing comparisons between crown materials and time points [11].

Data analysis

Data were analyzed using the IBM SPSS Statistics for Windows, version 20 software (Released 2011; IBM Corp., Armonk, New York, United States). Normality was confirmed using the Shapiro-Wilk test (p > 0.05). Paired tests were employed in this split-mouth study. Repeated measures analysis of variance (ANOVA) was used to compare longitudinal changes within groups (baseline, one month, and six months), while paired t-tests were used to analyze intergroup differences. All tests were two-tailed, with significance set at P < 0.05.

## Results

The study included 20 patients comprising 12 (60%) male and eight (40%) female participants with a mean age of 40.7 ± 6.09 years. Age distribution was similar between sexes (male: 40.58 ± 5.36 years; female: 40.88 ± 7.24 years). Balanced age and sex representations suggested minimal demographic bias (Table 1).

**Table 1:** Demographic details of study population. Age is represented as mean and standard deviation, and distribution of sex has been represented as frequency (n) and percentage (%), where n denotes number of patients.

Parameters	Male	Female	Total
n (%)	12 (60%)	8 (40%)	20 (100%)
Age (years), mean ± SD	40.58 ± 5.36	40.88 ± 7.24	40.7 ± 6.09

The baseline comparison between metal and zirconia crowns revealed no statistically significant differences in the clinical or microbial parameters. Plaque index scores (metal: 0.61 ± 0.14; zirconia: 0.55 ± 0.14; p = 0.184) and probing depth (metal: 1.45 ± 0.19 mm; zirconia: 1.44 ± 0.20 mm; p = 0.936) were comparable. Microbial analysis showed similar baseline levels of *S. mutans* (metal: 1.15 ± 0.64 x 10⁴ CFU/mL; zirconia: 1.12 ± 0.42 x 10⁴; p = 0.131) and* Lactobacillus *spp. (metal: 0.75 ± 0.32 x 10³; zirconia: 0.61 ± 0.28 x 10³; p = 0.367). The narrow 95% confidence intervals (CIs) crossing zero for all mean differences confirmed the balanced baseline characteristics (Table 2).

**Table 2:** Baseline characteristics of variables in the study population. p > 0.05 denotes no statistical significance using paired t-tests, CFU: Colony forming units.

Variables	Metal Crown (n=20), mean ± SD	Zirconia Crown (n=20), mean ± SD	Mean difference	95% Confidence Interval		t value	p-value
				Lower limit	Upper limit		
Plaque index	0.61 ± 0.14	0.55 ± 0.14	0.06	-0.03	0.15	1.35	0.184
Probing depth (mm)	1.45 ± 0.19	1.44 ± 0.20	0.01	-0.12	0.13	0.08	0.936
*Streptococcus mutans* (CFU X 10^4^/mL)	1.15 ± 0.64	1.12 ± 0.42	0.03	-0.29	1.08	1.32	0.131
*Lactobacillus spp.* (CFU x 10^3^/mL)	0.75 ± 0.32	0.61 ± 0.28	0.14	-0.51	1.15	1.22	0.367

Repeated measures analysis of variance (ANOVA) was conducted to compare the plaque index, probing depth, *S. mutans*, and *Lactobacillus *spp. within the metal and zirconia crown groups across three time points: baseline (T0), one month (T1), and six months (T2). In the metal crown group, significant increases were observed in all variables over time (p = 0.001). Similarly, in the zirconia crown group, all variables showed a significant increase from baseline to the six-month point (p = 0.001). These findings indicate that both crown materials experienced progressive changes in periodontal health and microbial colonization over the study period, with statistically significant differences within each group across time intervals (Table 3).

**Table 3:** Comparison of outcome variables at multiple time intervals within crown groups using repeated analysis of variance (ANOVA) test. *p < 0.05 denotes statistical significance CFU: colony-forming unit

Variables	Groups	Baseline (T0), mean ± SD	One month (T1), mean ± SD	Six months (T2), mean ± SD	F value	p-value
Plaque index	Metal	0.61 ± 0.14	2.01 ± 0.45	2.21 ± 0.47	171.13	0.001*
	Zirconia	0.55 ± 0.14	1.23 ± 0.38	1.33 ± 0.31	60.81	0.001*
Probing depth (mm)	Metal	1.45 ± 0.19	1.57 ± 0.22	3.12 ± 0.58	165.9	0.001*
	Zirconia	1.44 ± 0.2	1.48 ± 0.19	2.70 ± 0.46	110.84	0.001*
*Streptococcus mutans* (CFU X 10^4^/mL)	Metal	1.15 ± 0.64	2.85 ± 0.64	5.92 ± 0.89	299.78	0.001*
	Zirconia	1.12 ± 0.42	1.42 ± 0.42	3.66 ± 0.63	217.02	0.001*
*Lactobacillus spp. * (CFU x 10^3^/mL)	Metal	0.75 ± 0.13	1.22 ± 0.32	5.03 ± 0.64	584.38	0.001*
	Zirconia	0.61 ± 0.18	0.91 ± 0.28	2.06 ± 0.44	120.62	0.001*

Intergroup comparison revealed statistically significant differences in all measured parameters between the metal and zirconia crowns (p < 0.05). Metal crowns demonstrated greater deterioration than zirconia crowns, with higher mean changes in plaque index (p = 0.001), probing depth (p = 0.021), *S. mutans* (p = 0.004), and *Lactobacillus spp. *(p = 0.001). Notably, the largest disparity was observed in *Lactobacillus spp.* accumulation (mean difference = 2.67), suggesting that metal crowns may create a more favorable environment for acidogenic bacteria than zirconia crowns (Table 4).

**Table 4:** Comparison of change in outcome variables between metal and zirconia crowns using paired t-test. *p < 0.05 denotes statistical significance CFU: colony-forming unit

Variables (T2-T0)	Groups	Mean ± SD	Mean difference	95% Confidence Interval		t value	p-value
				Lower limit	Upper limit		
Plaque index	Metal	1.60 ± 0.50	0.82	0.54	1.1	5.91	0.001*
	Zirconia	0.78 ± 0.37					
Probing depth (mm)	Metal	1.67 ± 0.57	0.41	0.07	0.76	2.4	0.021*
	Zirconia	1.26 ± 0.52					
*Streptococcus mutans* (CFU X 10^4^/mL)	Metal	3.07 ± 1.02	0.83	0.28	1.38	3.03	0.004*
	Zirconia	2.24 ± 0.68					
*Lactobacillus *spp. (CFU x 10^3^/mL)	Metal	3.82 ± 0.73	2.67	2.26	3.08	13.25	0.001*
	Zirconia	1.15 ± 0.52					

## Discussion

The results of the present study demonstrated that both crown materials experienced significant increases in plaque index, probing depth, *S. mutans*, and *Lactobacillus *spp. from baseline (T0) to six months (T2) within each group. However, intergroup comparisons revealed that stainless steel crowns exhibited significantly greater increases in these parameters than zirconia crowns, with the most pronounced difference in *Lactobacillus *spp.colonization. These findings suggest that zirconia crowns may be less conducive to microbial colonization, particularly for acidogenic bacteria, than stainless steel crowns in root canal-treated teeth. The baseline data confirmed no significant differences between the metal and zirconia groups for the various parameters, indicating a balanced starting point. This was critical for split-mouth design, which minimized inter-patient variability by placing both crown types in the same oral environment [12].

The intergroup differences highlight the superior performance of zirconia in resisting microbial colonization. The greater increase in *S. mutans* and *Lactobacillus spp.* on stainless steel crowns aligns with prior research indicating that metal surfaces, owing to their higher surface roughness and free energy, promote bacterial adhesion more than ceramics [6,7]. The polished surface and lower surface free energy of zirconia likely contributed to its reduced microbial load, supporting its biocompatibility in the oral environment [6]. Souza et al. reported reduced bacterial adhesion after 48 hours in zirconia crowns [13]. In contrast, according to Metwally et al., the surface roughness of Bioflx crowns is comparable to that of stainless-steel crowns [14]. This difference could have been due to the material properties of Bioflx, which combines the qualities of zirconia and stainless steel.

Scheuerman et al. posited that irregularities present on polymeric surfaces facilitate bacterial attachment and biofilm formation, in contrast to ultra-smooth surfaces that do not support such processes [15]. When glazed and polished, zirconia exhibits superior characteristics compared to stainless steel crowns, thereby inhibiting microbial adhesion [6]. Myers et al. indicated that plaque formation occurs readily on the surfaces of stainless steel crowns, irrespective of the polishing techniques employed [16]; hence, it is imperative to prioritize oral hygiene practices to reduce plaque accumulation. This phenomenon may be attributed to physicochemical interactions involving electrostatic and van der Waals forces between the restorative surface and the microbial entities.

The significant increase in plaque index and probing depth in the metal group suggests that stainless steel crowns may exacerbate periodontal inflammation over time. This is consistent with a study by Adamczyk and Spiechowicz, who noted that metal restorations, particularly stainless steel, can accumulate more plaque owing to surface irregularities, leading to gingival irritation [17]. In contrast, the smoother margins of zirconia are likely to minimize plaque retention and reduce periodontal stress [6]. Similar results were reported by Mathew et al., who observed less plaque accumulation in zirconia crowns [7]. The study’s rigorous marginal adaptation checks, using light-body polyvinyl siloxane putty for impressions, ensured that differences in microbial load were primarily due to material properties rather than poor fit.

The pronounced difference in *Lactobacillus *spp. colonization is particularly noteworthy as these bacteria are strongly associated with caries progression in root canal-treated teeth. The qPCR method used in this study with species-specific primers provided high sensitivity and specificity, as supported by Yoshida et al., who validated qPCR for quantifying cariogenic bacteria [11]. The significant increase in *Lactobacillus *spp. on stainless steel crowns suggests that metal surfaces may create a more acidic microenvironment, favoring acidogenic bacteria. This aligns with a previous review that reported that *Lactobacillus *spp. thrives in low-pH environments [18], which may be exacerbated by plaque accumulation on rougher metal surfaces.

The preliminary phase of microbial colonization by an organism entails adherence to the host interface, where a higher free energy of the surface correlates with increased adhesion of microorganisms. Conversely, surfaces exhibiting greater hydrophilicity are expected to exhibit reduced microbial adherence [9]. *S. mutans* exhibits an elevated surface free energy and demonstrates a marked affinity for binding to surfaces characterized by a high free energy, such as stainless steel [19]. The initial adhesion and retention of *S. mutans *transpire through van der Waals attractive forces and electrostatic repulsive interactions with the crown surface. Furthermore, bacterial presence on uneven surfaces of prostheses affords greater protection against shear forces, thus enabling prolonged direct contact and fostering an alteration in the oral ecological landscape [20]. Similarly, *Lactobacillus *spp. exhibit strong adhesion to surfaces with high roughness and surface free energy, such as stainless steel, owing to their ability to form robust biofilms in acidic microenvironments. The adhesion of *Lactobacillus *spp. is facilitated by hydrophobic interactions and extracellular polysaccharide production, which enhances their retention on irregular surfaces, promoting sustained colonization and contributing to the ecological shift toward a cariogenic oral environment [21,22].

Clinical implications

These results suggest that zirconia crowns may be preferred over stainless steel for root canal-treated teeth, particularly in patients prone to caries or periodontal issues, due to their lower susceptibility to *S. mutans* and *Lactobacillus *spp.colonization. Clinicians should consider zirconia for posterior molars, where aesthetics are less critical in reducing microbial-related complications. The significant periodontal deterioration (increased probing depth and plaque index) with stainless steel crowns underscores the need for enhanced oral hygiene protocols in patients receiving metal restorations. Regular prophylaxis and patient education on low-sugar diets and flossing, as implemented in this study, are essential for mitigating these risks. The use of qPCR for microbial analysis offers a precise tool to monitor restoration biocompatibility in future clinical studies.

Limitations

This study has several limitations. The non-randomized design, based on clinical indications and patient preference, may have introduced a selection bias, although the split-mouth approach mitigated this by controlling for intra-patient variability. The sample size was sufficient based on power calculations, but it may limit the generalizability to diverse populations. The six-month follow-up period provided valuable insights but may not capture long-term microbial or periodontal changes. Additionally, although qPCR was highly sensitive, it did not differentiate between viable and non-viable bacteria, potentially overestimating the microbial load. This study focused solely on *S. mutans* and *Lactobacillus *spp., excluding other oral pathogens that may influence the outcomes. Finally, patient compliance with oral hygiene and dietary instructions relied on self-reports, which may have introduced reporting bias.

## Conclusions

This study demonstrated that zirconia crowns exhibited significantly lower microbial colonization by *S. mutans* and *Lactobacillus *spp. compared to stainless steel crowns in root canal-treated teeth over a six-month period. Both crown materials showed progressive increases in microbial load, plaque accumulation, and periodontal changes from baseline to six months, but the extent of these changes was notably greater in stainless-steel crowns. These findings suggest that the smoother surface and lower surface free energy of zirconia crowns contribute to reduced bacterial adhesion, making them a more biocompatible option for restorations in root canal-treated teeth. These results highlight the importance of material selection for minimizing microbial-related complications and support the preference for zirconia crowns to enhance clinical outcomes in restorative dentistry.
